# An Improved Method of Taking Plaster Impressions

**Published:** 1875-06

**Authors:** J. Smith Turner

**Affiliations:** London, England.


### SELECTED ARTICLES.

---

### ARTICLE VII.

### *An Improved Method of Taking Plaster Impressions.*

BY J. SMITH TURNER, M. R. C. S., LONDON, ENGLAND.

I beg to thank you for the honor you have conferred upon me in asking me to contribute a paper to your transactions. I trust this communication will be accepted by you in the spirit in which it is sent; more as an acknowledgement of the compliment you have paid me than for any intrinsic merit it may possess.

The question of plaster impressions has little novelty to recommend it. Like many other questions in our profession, it comes to the front, or falls to the rear, according to the difficulties which beset the more active minded among the rising and the more recently-established members of the profession. The senior members, who have overcome, or think they have overcome, all the difficulties attending any process, are generally contented to let matters rest. They can accomplish all they desire by a system which has become familiar to them by practice, and to the deficiencies of which (this I say with all respect) they have learned to shut their eyes; or if they see them, it is in a light flattering to their own proficiency, which enables them to give a pharisaiacal hug to their superiority. The young members, on the other hand, who are ever looking out for a royal road to excellence, make a rush at the last new thing perhaps to discard it with equal readiness. Plaster impression-taking has gone through those phases, and is likely, in common with other questions, to go through the same again.

There are those who use one material, and those who use another; and there is a large number of men who, while believing in the superior excellence of plaster moulds for almost every case, confess to a want of manipulative skill, or to a want of power over their patients which deprives them, in many instances, of the use of this excellent material. There are also those who timidity deters them from ever commencing its use, while many have yet to learn how to use the more simple materials. It is to the timid and unskillful, with whom I have that fellow-feeling which "makes us wondrous kind," that I would now address myself. If proficients in the art see anything worthy of notice in my plan, I shall be all the better pleased. I am not going to argue the question, nor enumerate the difficulties connected with the subject. Suffice it to say, that objections are raised to plaster moulds for partial cases, owing to the difficulty of obtaining a correct model all round on which teeth can be mounted as well as a plate fitted. To get a good mould involves more endurance than many patients can give, and a great amount of work from the operator, both in breaking up the mould and in piecing it together again.

I propose, by making certain alterations in the tray, and by applying the plaster in a different manner than hitherto, to obviate some of these disadvantages. I convert the handle of the impression tray into a funnel, which opens by an oval aperture on the base plate or palate of the tray, immediately under its external aspect, the lower border of which forms the upper boundary of the opening. The outer end of the handle forms the mouth of the funnel, and this has a rim about the twelfth of an inch wide round the outer side of its free border.

I take an impression in the ordinary way, with wax, but giving no heed to the palate, direct my attention to the outer circumference of the mouth, or, in fact, to every part on which I do not wish the plate to fit.

When I withdraw this preliminary mould, I cut away the wax from wherever I wish my plate to rest, as these are the parts of which I wish a plaster impression. I have then left a wax mould of the front of the remaining teeth and gums, and also of a part of the palate behind that on which I wish the posterior part of the projected plate to rest. My next step is to make air-vents round the margin of the wax. I then oil the funnel, and fix to its mouth, by an elastic band or a piece of string, a small pouch composed of sausage-skin, or some such cheap, yielding, waterproof material; then mixing my plaster in the usual way, I pour it into the pouch through the palatine opening of the funnel The tray is then replaced in the mouth, and held in position by the left hand, and with the right the plaster is pressed out of the pouch, and when it passes through the air-passages with a bubbling sound, you may reckon enough has been squeezed in, and that the impression is taken. The plaster soon became hard enough to admit of the withdrawal of the hand which holds the pouch. An impression thus taken is easily withdrawn from the mouth, owing to the yielding nature of the wax, while all essentials are preserved by the plaster. If a fracture occurs, the plaster is so thick that it is easily pieced. By this process there is no chance of any but the smallest gushing of plaster, which may pass through the air-vents getting where it is not wanted. The vents should be made free and wide where they open between the margin of the wax and the cheek or lip, but those in the bar of wax which stretches across the posterior part of the palate should be narrow and V-shaped, the apices resting on the tray. Of these last vents two are sufficient.

I send a model taken in this way, and a tray prepared for taking the same, and if my paper does not prove practical, I hope it may prove suggestive. I may add, that the plaster is best mixed with tepid water as a hardening medium. If a saline be used, it makes the plaster unpleasantly cold.—*N. Y. Odontological Society Transactions.*

